# Progress in Medicine

**Published:** 1895-06-22

**Authors:** 


### PROGRESS IN MEDICINE.

### DISEASES OF CHILDREN.

(*Continued from page* 185.)

**The Resuscitation of Still-born Infants.**—At a discussion on this subject at the Academy of Medicine, Dr. Pinard[16] reported the results of treatment in fifty cases, of whom forty-four were resuscitated by various measures. Nineteen subsequently died, and the remaining twenty-five left the hospital in good condition. Two methods of treatment were employed—the one a combination of insufflation and flagellation, and the other Laborde's method of rhythmical traction on the tongue. He concluded that at present there was no evidence that the tongue method was superior to that of insufflation. Dr. Laborde pointed out that to estimate properly the value of the tongue method, it was necessary to continue the process for at least ten minutes. His own experience was that this procedure had often succeeded after all other methods had failed.

**Erythema Urticatum.**—Funk and Grundzach[17] have drawn attention to the connection that exists between erythema urticatum, rickets, and dilatation of the stomach. The skin lesion is situated for the most part on the extensor surfaces of the extremities, and on the back of the hands and feet. Vesicles and blebs, accompanied by œdema, may take the place of wheals. The primary disease is rickets, which leads to dilatation of the stomach, and that again is followed by the development of urticaria. In forty-five cases of erythema urticatum in children under two years of age the ordinary symptoms of rickets were invariably present. The accompanying digestive troubles were colic and constipation, and great thirst was frequently noted. They observed that a certain family "disposition" was often present in the subjects of erythema urticatum.

**Ear Disease in Childhood.**—In a clinical lecture on this subject Dr. Woakes[18] lays stress on the influence of teething in the production of otitis, and advocates free incision of the swollen gums in such cases. The mechanism by which this is brought about is a nervous one, dental irritation causing an impulse to pass along the inferior dental nerve, through the otic ganglion, to the tympanic branch of the carotid artery. That vessel becomes dilated, and effusion, transudation of cytoblasts, and the formation of pus follow. Further dangers lie ahead from the fact that the tympanum often communicates directly with the brain by a prolongation of the dura mater passing through the squamo-petrosal suture, and also by means of the tympanic branches of the middle meningeal artery. He recommends, for diagnostic purposes, the use of an anæsthetic, so as to allow of a thorough examination of the ear. In early acute cases the application of one or more leeches to the concha or the mastoid process gives the speediest relief. Bromide of potassium and tincture of aconite are useful medicines. Serious complications are indicated by a persistent rolling of the head, pointing to labyrinthine mischief from pressure of the fluid. Relief must then be sought by puncturing the membrane, and by irrigation of the ear and post-nasal space with a warm alkaline lotion, namely, half an ounce of bicarbonate of soda and half a drachm of carbolic acid in a pint of water.

**Latent Hereditary Syphilis.**—The diagnosis of this condition in young infants is sometimes difficult from the absence of any visible signs of the disease. Dr. H. Pouzol[19] observes that in such cases valuable information may be gained by the study of the progressive loss of weight unaccompanied by any marked digestive disturbances. During the first two days of life an infant loses weight, but with proper nursing this loss is recovered in from six to seven days. A syphilitic child, on the other hand, does not gain weight, but continues to lose flesh steadily, and the occurrence of this condition without any other apparent cause points strongly to syphilitic disease, and suggests the immediate use of full doses of mercury.

**The Treatment of Whooping Cough.**—Professor Hollopeter[20] considers that in the earlier stages of whooping cough a sign of considerable diagnostic value is the peculiar puffiness of the eyes, and the swollen or œdematous condition of the whole face. As regards treatment, he recommends tincture of belladonna in one-drop doses for every month of the child's life, and increased doses, if necessary, until physiological effects are produced; the application of a belladonna plaster to the interscapular region, and the use of a peroxide of hydrogen spray to the throat, with or without the addition of cocaine. Truwald[21] recommends antispasmine in this disorder as the result of his experience in two hundred cases. The form employed is a 5 or 10 per cent. solution in cherry laurel water, of which three or four minims may be given three times a day to an infant under six months; five to ten minims to a baby between six and twelve months, and so on according to age. Dr. A. Vignol[22] advises a mixture of belladonna and aconite before meals, and after fifteen days of this treatment the use of potassium bromide. Dr. Rehn[23] has used antipyrin mandelate (a compound of antipyrin and mandelic acid) in fifty cases of whooping cough. He claims that it diminishes the frequency of the paroxysms and the vomiting, improves the appetite, and reduces the duration of the disease in ordinary cases to three weeks. Similar good results have been obtained from the employment of bromoform by Dr. Carpenter.[24] Care must be taken to prevent the occurrence of overdosing, of which the symptoms are pallor, staggering, dilatation of the pupils, coma, heart failure, and collapse. He recommends one to two drops three times a day for infants, and to patients over two years of age two to five drops. Dr. Baron,[25] in accordance with the teaching of Binz and Ungar, believes that better results are obtained in whooping cough from quinine than from any other drug.

**The Feeding of Infants.**—Professor Heubner[26] regards cows' milk as the only substitute for mothers' milk in the rearing of infants. Yet in a certain number of cases cows' milk fails, and the infant passes through a series of digestive disturbances, and finally dies from intestinal intoxication. Various causes have been assigned for this failure, amongst others the indigestibility of the milk albumens, and more recently the bacteria in the milk. He considers that the latter point has not yet been definitely settled, but thinks that in the meantime precautions ought to be taken to ensure for the supply of large cities milk drawn under aseptic conditions, kept in aseptic receptacles, and carried as short distances as possible. Gärtner[27] recommends for infants a "fat milk" food, prepared from diluted cows' milk by means of a centrifugal separator, and claims that this resembles human milk very closely. Flügge[28] discusses at length the bacteria of milk, their effects, and the best means of preventing their development. The elaborate measures employed are somewhat difficult to carry out successfully amongst those who suffer most from impure milk. As the result of a series of experiments Heubner[29] has come to the conclusion that even very young infants can digest farinaceous food as well as albumen and fats. He advises the use of rice or banana flour when digestive disturbances are present as a means of preventing irritation of the intestinal epithelium.

**Renal Diseases.**—Dr. G. W. Lueck[30] regards pilocarpine as the most beneficial drug in renal dropsy, and describes five illustrative cases. He sums up the treatment as follows: Absolute rest in bed, the administration of pilocarpine (gr. 1-20th every three or four hours for a child of ten years) and alkaline drinks, and the application of hot flaxseed and mustard poultices over the region of the kidneys to aid the action of the pilocarpine. Dr. Robert Turner[31] has described three cases of infantile diarrhœa complicated with acute nephritis. The patients were all rachitic, and the diarrhœa at first presented no unusual symptoms, but later on renal symptoms supervened, and death followed from uræmia in every case. The possibility of a scarlatinal origin was excluded, and no other cause was detected, so that the writer is at a loss to explain the etiology of these cases, but suggests that there may have been absorption from the intestines of ptomaines and alkaloides, which in the process of excretion by the kidneys produced inflammatory disease in these organs. Dr. Emmet Holt[32] has met with three cases of acute pyelitis in infants, of which the signs were pyrexia and pyuria. There was no evidence of malaria, and quinine did not seem to have much influence on the course of the disease, which in all the cases ended in recovery. Influenza might have been a factor in one case. There was no evidence of vulvo-vaginitis.

**Standards of Health.**—Dr. J. Lewis Smith[33] has investigated the subject of the temperature in healthy children. In the case of infants under six months of age, the rectal temperature averaged 98 deg. Fahr., and the axillary 98·3 deg.; from six to twelve months, rectal 98·6 deg., axillary 98·3 deg.; from twelve to eighteen months, rectal 98·4 deg., axillary 98·3 deg.; from eighteen to thirty months, rectal 98·9 deg., axillary 98·1 deg. Sudden and marked rises follow from slight causes, such as mental excitement, indigestion, or constipation. As regards the pulse rate and respirations he gives the following averages: Under six months, 125 and 44·8; from the sixth to the twelfth month, 124 and 34·8; from the twelfth to the eighteenth month, 115·5 and 35·4; and from the eighteenth to the thirtieth month, 111·8 and 29·8. Taking 200 infants who presented no signs of rickets, he found that the dates of eruption of the first teeth were as follows: in three infants at two months, in twenty at five months, in twenty-four at six months, in thirty-seven at seven months, in twenty-eight at eight months, in twenty at nine months, in fourteen at ten months, in fifteen at eleven months, in eight at twelve months, and in one at thirteen months.

**Intubation of the Larynx.**—Dr. George Bieser[34] has performed intubation in twenty-two cases of diphtheritic or membranous laryngitis, of which number eight, or thirty-six per cent., recovered. He prefers intubation to tracheotomy because the former (1) is simpler and more rapid, (2) is preferred by parents, (3) requires no special assistance, (4) can be employed without an anæsthetic, (5) is less liable to cause shock and hæmorrhage, (6) allows of efficient coughing afterwards, (7) leads to simpler after treatment, and (8) leaves no scar. His conclusions as to treatment are as follows: Relieve stenosis by intubation or calomel fumigation, guard against cardiac failure by absolute rest in bed, and the early and free administration of alcohol; if the child does not struggle, irrigate the throat and nares; if it does, trust to internal disinfectants, mercury, iron, and chlorate of potash; give stimulants before and after removing the tube.

[16] The Med. Week, Jany. 18 and 25, 1895. [17] Monats f. prakt. Dermat., Bd. XVIII., No. 3, and Med. Chron., Nov. 1894. [18] Clin. Jour., Nov. 21, 1894. [19] Grad. Thesis, Paris, 1894, and Med. Week, Nov. 30, 1894. [20] Therap. Gaz., Oct. 15, 1894. [21] Clin. Jour., March 6, 1895. [22] Jour. des Pratic., 1894, No. 4, p. 44, and Am. Jour. Med. Sci., Dec., 1894. [23] Münch. Med. Woch, Nov. 13, 1894, and Ep. B. M. J., Dec. 8, 1894. [24] Polyclinic, June 16, 1894, and Therap. Gaz., Oct. 15, 1894. [25] Berlin Klin. Woch, 1893, No. 48. [26] Med. Week, Sep. 21, 1894. [27] Allg. Wien. Med. Zeit., Oct. 13, 1894, and Ep. B. M. J., Nov. 10, 1894. [28] Zeitsch. f. Hyg., 1894, XVII., 272, and N. Y. Med. Jour., Dec., 1894. [29] Med. Week, Jan. 25 1895. [30] Therap. Gaz., Nov. 15, 1894. [31] The Pract., Oct., 1894. [32] N. Y. Med. Jour., Oct. 20, 1894. [33] N. Y. Med. Rec., Jany. 26, 1895. [34] Arch. of Paediat, Feb., 1895.

